# Changes in dietary fiber intake in mice reveal associations between colonic mucin *O*-glycosylation and specific gut bacteria

**DOI:** 10.1080/19490976.2020.1802209

**Published:** 2020-09-29

**Authors:** Hasinika K. A. H. Gamage, Raymond W. W. Chong, Daniel Bucio-Noble, Liisa Kautto, Anandwardhan A. Hardikar, Malcolm S. Ball, Mark P. Molloy, Nicolle H. Packer, Ian T. Paulsen

**Affiliations:** a ARC Industrial Transformation Training Centre for Molecular Technologies in the Food Industry, Macquarie University, Sydney, Australia; b Department of Molecular Sciences, Macquarie University, Sydney, Australia; c Islet Biology and Diabetes, Faculty of Medicine and Health, The University of Sydney, Sydney, Australia; d Gratuk Technologies Pty Ltd, Sydney, Australia

**Keywords:** gut microbiota, mucin *O-*glycosylation, glycan-microbiota interaction, dietary intervention, dietary fiber

## Abstract

The colonic mucus layer, comprised of highly *O-*glycosylated mucins, is vital to mediating host-gut microbiota interactions, yet the impact of dietary changes on colonic mucin *O-*glycosylation and its associations with the gut microbiota remains unexplored. Here, we used an array of omics techniques including glycomics to examine the effect of dietary fiber consumption on the gut microbiota, colonic mucin *O-*glycosylation and host physiology of high-fat diet-fed C57BL/6J mice. The high-fat diet group had significantly impaired glucose tolerance and altered liver proteome, gut microbiota composition, and short-chain fatty acid production compared to normal chow diet group. While dietary fiber inclusion did not reverse all high fat-induced modifications, it resulted in specific changes, including an increase in the relative abundance of bacterial families with known fiber digesters and a higher propionate concentration. Conversely, colonic mucin *O*-glycosylation remained similar between the normal chow and high-fat diet groups, while dietary fiber intervention resulted in major alterations in *O*-glycosylation. Correlation network analysis revealed previously undescribed associations between specific bacteria and mucin glycan structures. For example, the relative abundance of the bacterium *Parabacteroides distasonis* positively correlated with glycan structures containing one terminal fucose and correlated negatively with glycans containing two terminal fucose residues or with both an N-acetylneuraminic acid and a sulfate residue. This is the first comprehensive report of the impact of dietary fiber on the colonic mucin *O-*glycosylation and associations of these mucosal glycans with specific gut bacteria.

## Introduction

The gut microbiota maintains a symbiotic relationship with the host and is essential for regulating host metabolism and immunity. A single layer of enterocytes separates the host from the colonic lumen. Thus, to prevent the translocation of commensal gut microorganisms and enteric pathogens across the large intestine epithelium, the host has developed multiple defense mechanisms including the mucus layer, which serves as a physical barrier and an important mediator of host-gut microbiota interactions.^1–3^ This mucus layer is comprised of two discrete layers; an inner layer that is largely devoid of microorganisms, and an outer layer that is loosely attached and inhabited by commensal gut microorganisms.^4^

The colonic mucus layer is primarily composed of mucin-2-glycoprotein (MUC2), a highly glycosylated protein that contains abundant amounts of the hydroxy amino acids, serine (Ser), and threonine (Thr), that act as attachment sites for hydroxy linked carbohydrate chains also known as *O*-linked glycans.^5,6^ These *O*-linked glycans contribute to at least 80% of the total molecular weight of the colonic mucus layer and are responsible for many biological and physical properties such as bacterial adhesion, viscosity, and water-binding capacity.^1,6,7^ Mucin *O*-glycosylation starts with the attachment of GalNAc residues to the hydroxyl group of Ser and Thr residues on the protein backbone, which are elongated into four different core structures.^8^ These core structures are further elongated by the addition of N-acetylgalactosamine (GalNAc), galactose (Gal), and N-acetylglucosamine (GlcNAc) and are commonly terminated by sulfate, fucose (Fuc), and N-acetylneuraminic acid (Neu5Ac) residues. Inter-species differences in the abundance of core structures have been observed, with Core 1 and 2 structures predominant in mice while Core 3 and 4 structures predominant in humans. Studies in both humans and mice have shown glycan gradients along the gastrointestinal tract. For example, more acidic glycans, such as those containing Neu5Ac, are more abundant in the proximal end of the mouse gastrointestinal tract while Fuc tends to be more abundant in the distal end.^3,9^ In contrast, humans have more Fuc in the proximal end of the gastrointestinal tract while acidic glycans are more abundant in the distal end.^2,10^ This regiospecific distribution suggests that specific glycans may be important for the selection of microbiota along the gastrointestinal tract.

The colonic mucin layer and gut microbiota maintain bidirectional interactions that are essential for the development of the mucus layer and microbial colonization in the gut. As observed through histochemical studies, the colonic mucus layer of germ-free rats is thinner and less compact compared to conventionally raised rats, suggesting an essential role of the gut microbiome in the maturation of the colonic mucus layer to its full functional potential.^6,11^ Mono-colonization studies in gnotobiotic mice have suggested that the commensal gut bacteria *Bacteroides thetaiotaomicron* and *Faecalibacterium prausnitzii* can modulate mucus production by assisting goblet cell differentiation and regulating the expression of genes involved in mucin glycosylation.^12^ Gut microbial metabolites such as acetate also have been proposed to regulate colonic mucin *O*-glycosylation through their impact on the expression of host glycosyltransferases.^12–14^ Germ-free mice, which lack microbial metabolites including acetate, have been shown to contain shorter mucin *O*-glycans and this correlated with lower expression levels of specific host glycosyltransferases.^13,14^ Concurrently, the colonic mucin glycans play a key role in selecting microbial communities along and across the gastrointestinal tract by providing adhesion sites and serving as a nutritional source for specific gut bacteria.^15^ The cell surface adhesins, lectins, glycan receptor proteins, and carbohydrate-active enzymes present in the gut microbiome facilitate bacterial adhesion, recognition, and digestion of mucin glycans. However, only a few intestinal bacteria, such as *Akkermansia muciniphila, Bacteroides thetaiotaomicron, Bifidobacterium bifidum, Bacteroides fragilis*, and *Ruminococcus gnavus* have been studied for their ability to recognize and hydrolyze mucin oligosaccharide chains.^3,16-20^

Degradation of mucin glycans is possible through the activity of bacterial glycoside hydrolases that remove monosaccharides in a stepwise manner.^21^ This sequential breakdown of glycans leads to thinning of the mucus layer resulting in contact between the gut microbiota and host, leading to conditions such as ulcerative colitis, colorectal cancer, and infections.^5,22,23^ The gut microbiota degrades colonic mucin glycans and utilizes the saccharides as a nutrient source particularly in conditions of limited dietary intake. For example, studies in gnotobiotic mice have shown that specialized commensal bacteria selectively degrade mucin glycans in response to low levels of dietary fiber.^24^ Previous studies have also shown that dietary fiber inclusion can induce changes in mucin glycosylation.^25–27^ However, due to limitations of the techniques used, these studies could only distinguish changes in acidic, neutral, or fucosylated glycans and lacked detailed structural characterization of the gut mucin glycans.

While a plausible link between mucin glycosylation and the gut microbiota is being established, there is still limited information on how specific microbes interact with specific mucin glycans within the complex system of the gut and the effects of dietary intake. In this study, we used NutriKane™, a fiber product derived from sucrose-removed sugarcane, and Benefiber®, a wheat dextrin, as dietary interventions in high-fat diet-fed C57BL/6J mice. Both NutriKane and Benefiber have been studied as dietary fiber^28^ and a modulator of the gut microbiota and short-chain fatty acid (SCFA) production *in vitro*, ^29^ NutriKane has also been investigated for its anti-inflammatory properties.^30^ Here, we examined the effect of dietary changes on colonic *O*-glycosylation profiles and associations between mucosal *O*-linked glycans and the gut microbiota using a comprehensive multi-omic-based study approach.

## Results and discussion

### Dietary fiber modifications did not significantly alter the host physiology

C57BL/6J mice were randomized into four groups and given a normal chow (NC), high-fat diet (HF), high-fat diet modified with NutriKane (HF-NK) or high-fat diet modified with Benefiber (HF-BF); the detailed experimental design is provided in Figure S1 and nutritional composition of each treatment diet is provided in Table S1. Host physiology in terms of glucose tolerance (Figure S2A); plasma markers of diabetes and obesity (GLP1, insulin, and PAI1, Figure S2B-D; other markers, Figure S3); plasma cytokines (IL-1β and GM-CSF, Figure S2E-F; other markers, Figure S3); liver proteome (Figure S2G and Table S2) and cecum mass (Figure S4A), which has been shown to decrease with lower dietary fiber intake, ^31,32^ demonstrated significant differences between the NC and HF groups. The fiber-supplemented HF-NK or HF-BF groups showed no significant differences compared to the HF group indicating no significant impact as a result of dietary fiber modification. Compared to the NC group, the HF, HF-NK and HF-BF groups exhibited significantly lower (*P* < .0001) feed consumption (Figure S5A), though the calculated energy intake (Figure S5B) and body weight (Figure S5C) showed no significant difference across the four groups.

### Addition of dietary fiber altered the gut microbiota composition and SCFA production

**Figure 1.**
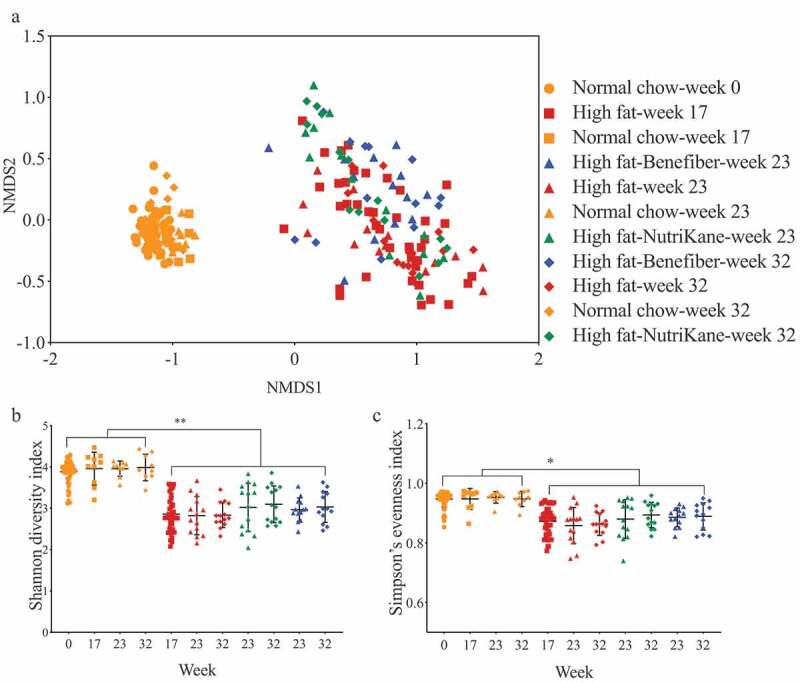
Ordination and alpha diversity of the gut microbiota. Data shown for mice fed each of the four diets at weeks 0, 17, 23 and 32. (a) Ordination of the gut microbiota shown as a Bray-Curtis similarity-based nMDS plot. Gut microbial diversity and evenness shown as (b) Shannon diversity and (c) Simpson’s evenness indices, respectively. Mean values with ± SD are shown (** *P* < .01, * *P* < .05).

The impact of dietary interventions on the gut microbiota was determined by 16S rRNA gene amplicon sequencing. The overall gut microbiota structure in the NC group was significantly different (*P* < .0001, PERMANOVA) compared to the HF group (Figure 1a). While the transition to HF-NK or HF-BF did not significantly alter the overall microbiota structure, there were specific operational taxonomic units (OTUs) in the HF-NK and HF-BF groups that were significantly different in abundance compared to the HF group. The alpha diversity, as determined by Shannon diversity and Simpson’s evenness indices, was significantly higher in the NC group compared to HF, HF-NK, and HF-BF groups, with no significant difference observed between HF, HF-NK, and HF-BF (Figure 1b,c).

**Figure 2.**
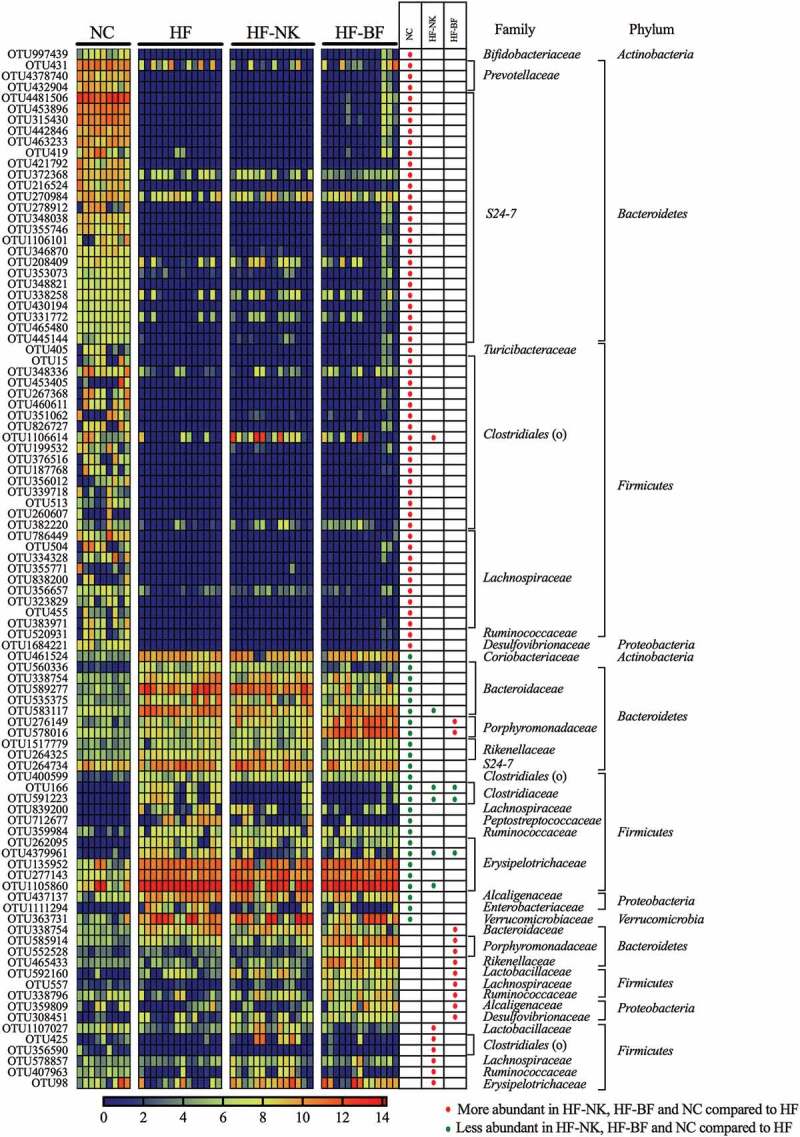
The relative abundance (Log_10_ transformed) of the OTUs that were significantly different between dietary groups (NC-normal chow, HF-high fat diet, HF-NK-high fat diet modified with NutriKane and HF-BF-high fat diet modified with Benefiber). The abundance of OTUs are shown per mouse. Differentially abundant OTUs were determined through LEfSe analyses between HF vs NC, HF vs HF-NK and HF vs HF-BF groups. Rows in the unpaired heatmap correspond to the abundance of the OTUs and columns correspond to individual mice in each dietary group. Red and blue denote the highest and lowest relative abundance, respectively, as shown in the legend. The changing direction of the relative abundance of the OTUs in comparison to the HF group is shown in the table, red and green dots denote more and less abundant compared to the HF group, respectively. The taxonomy of the OTUs (family and phylum) are shown on the right. The relative abundance of the these OTUs per mouse is provided in Table S3.

Linear discriminant analysis effect size (LEfSe) method was used to identify the bacterial families (Figure S6) and OTUs (Figure 2) that were altered by HF, HF-NK, or HF-BF. Comparing HF to NC, the relative abundance of 25 OTUs in the families *Erysipelotrichaceae, Bacteroidaceae, Alcaligenaceae, Clostridiaceae*, and *Verrucomicrobiaceae* was significantly higher. Concurrently, the relative abundance of 55 OTUs in the family S24-7, *Lachnospiraceae, Prevotellaceae*, and order *Clostridiales* was significantly lower in the HF group compared to the NC group. These HF-induced changes in the gut microbiota are consistent with several previous studies in mice, which have reported alterations in the abundance of these specific gut bacteria upon consumption of a high-fat diet.^33–36^

We then identified bacterial OTUs that were significantly different upon dietary fiber addition (Figure 2). Comparing HF-NK to HF, the relative abundance of seven OTUs in the families *Lactobacillaceae, Lachnospiraceae, Ruminococcaceae*, and order *Clostridiales* was significantly higher with NK addition. While the abundance of five OTUs in the families *Bacteroidaceae, Clostridiaceae, Lachnospiraceae*, and *Erysipelotrichaceae* was significantly lower. Compared to the HF group, the relative abundance of 11 OTUs in the families *Porphyromonadaceae, Bacteroidaceae, Rikenellaceae*, and *Lactobacillaceae* was significantly higher in the HF-BF group, whereas the relative abundance of three OTUs in *Clostridiaceae* and *Erysipelotrichaceae* was significantly lower. These OTUs that were highly abundant in the HF-NK and HF-BF groups belong to families with members associated with fiber digestion.^29,37-41^ The difference in the specific OTUs that were higher with each fiber addition may be due to variations in the chemical nature of NutriKane and Benefiber.^28,29^

As a major by-product of gut microbial fiber digestion, SCFAs are indicators of fiber accessibility and fermentation. Fecal concentrations of three main SCFAs, acetate, butyrate, and propionate were determined by gas chromatography coupled with flame ionization detection (GC-FID). In comparison to NC, the HF diet was associated with significantly lower concentrations of all three SCFAs (Figure S7). While HF-NK group showed no significant difference in the SCFA concentrations compared to the HF, HF-BF resulted in a significant increase in propionate concentration. This fiber product-specific impact on the production of SCFAs is likely due to variations in the fermentability of the two products. Benefiber contains a higher amount of soluble dietary fiber while NutriKane has a higher amount of insoluble dietary fiber.^29^ The gut microbiota rapidly digests soluble dietary fiber compared to insoluble fiber, likely contributing to the difference in SCFA production.

Correlation analysis revealed significant associations between the concentration of each SCFA and specific gut bacterial OTUs including those in the families S24-7, *Lachnospiraceae*, and order *Clostridiales* (Table S5). The concentration of propionate was positively associated (Pearson correlation > 0.38) with the abundance of bacterial OTUs within the family *Rikenellaceae* (OTU465433) and species *Parabacteroides distasonis* (OTU276149, OTU578016, OTU585914, and OTU552528), the abundance of these OTUs was significantly higher in the HF-BF group (Figure 2). These links are consistent with previous reports on the role of members in *Rikenellaceae* and *Parabacteroides distasonis* in propionate production.^42,43^

### *Addition of dietary fiber altered colonic mucus layer* O-*glycosylation*

**Figure 3.**
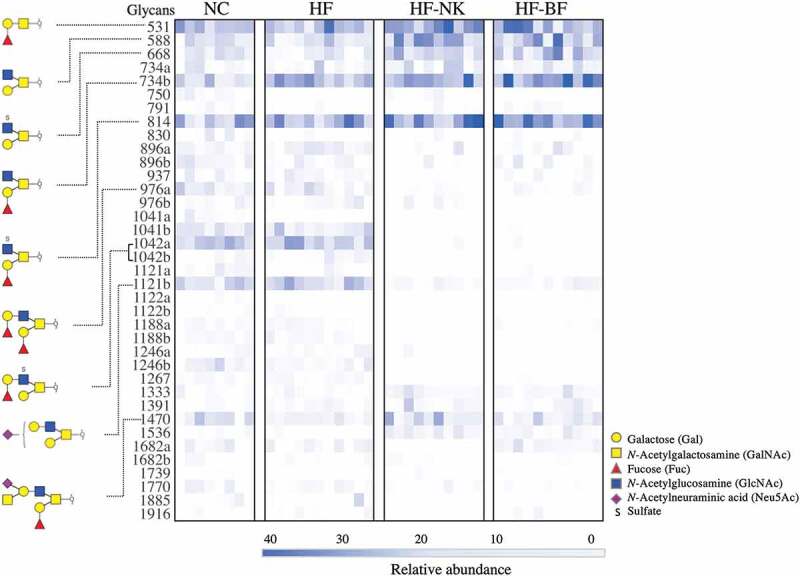
Unpaired heatmap illustrating the relative abundance of *O*-linked glycans. Glycans were released from the colonic mucin of mice given a normal chow (NC), high fat diet (HF), high fat diet modified with NutriKane (HF-NK), or high fat diet modified with Benefiber (HF-BF) diet. Glycans are listed in rows by their neutral molecular mass with structural isomers distinguished alphabetically. Each column represents a single mouse. Glycans with relative abundance significantly different between diets are presented on the left.

**Figure 4.**
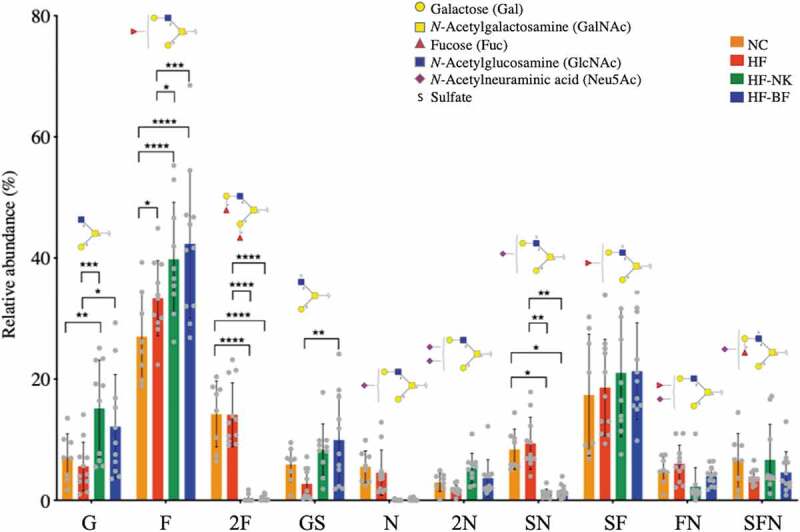
Effect of diet on the relative abundance of *O*-glycan structures on gut mucins. Glycans are grouped based on the type and presence of terminal residues: G- Gal, F- Fuc, 2F- double Fuc, GS-sulfate and Gal, N-Neu5Ac, 2N- double Neu5Ac, SN- sulfate and Neu5Ac, SF- sulfate and Fuc, FN- Fuc and Neu5Ac, and SFN- sulfate, Fuc and Neu5Ac. Statistically significant differences in glycan abundances between dietary groups (NC-normal chow, HF-high fat diet, HF-NK-high fat diet modified with NutriKane and HF-BF-high fat diet modified with Benefiber) were identified using two-way ANOVA with Tukey’s multiple comparison tests (****P < .0001, ***P < .001, **P < .01, *P < .05).

*O*-linked glycans released from colonic mucins were separated by porous-graphitized carbon liquid chromatography (PGC-LC) and analyzed by tandem mass spectrometry (MS/MS). A total of 37 unique *O*-glycan structures were detected and putatively determined by interpretation of the MS/MS fragmentation spectra (Figure 3 and Table S6). Mucin glycans contained Fuc (F), sulfate (S), and Neu5Ac (N) as terminal residues in varying combinations and degrees of substitution (Figure 4).

The majority of glycans detected were assigned as Core 2 branched structures (Galβ1-3(Galβ1-6) GalNAc) that are formed by the extension of Core 1 structures (Galβ1-3GalNAc) (Table S6). The only Core 1 glycan detected, glycan 531, while abundant in all samples, was significantly higher in relative abundance in HF-NK and HF-BF (Figure 3, Table S6). Similarly, the relative abundance of Core 2 glycans 588, 668, 734b, 814, and 1470, was significantly higher in HF-NK and HF-BF compared to HF, while the relative abundance of glycans 1042a, 1042b, and 1121a, was significantly lower (Figure 3, Table S6). Comparing the glycosylation profiles of NC and HF fed mice revealed striking similarities with only two glycans that were significantly different in relative abundance; the 734b glycan was more abundant in HF compared to NC, while 976a was less abundant in HF compared to NC. Overall, NC and HF groups were similar in mucin *O-*glycosylation profile to each other, as was glycosylation profiles in the HF-NK and HF-BF groups despite the chemical differences in the two dietary fiber products (Table S1). The nutritional composition of NC and HF differs dramatically, particularly the fat content (4.8% and 60% w/w, respectively), but both diets contain cellulose as a source of dietary fiber. This cellulose component in the HF was replaced with either NutriKane or Benefiber in HF-NK and HF-BF diets, respectively, the rest of the nutritional composition of these two diets was similar to the HF diet. Our results demonstrated similar glycosylation profiles in the HF group compared to the NC group, whereas there were major differences when compared to the HF-NK and HF-BF groups, potentially indicating a greater influence of dietary fiber than an increase in fat content on colonic mucin *O*-glycosylation. Examining the impact of these fiber products in a normal chow will provide further insight into the specific impacts of dietary fiber and fat content on mucin *O*-glycosylation.

Fucose, present as the blood group H antigen (Fucα1-2 Galβ1-), was an abundant terminal residue with approximately 60% of *O*-glycans detected containing at least one Fuc residue (Figure 4). This was consistent with previous studies showing fucosyltransferase 2 (Fut2), an enzyme responsible for attachment of α 1–2 linked Fuc, as the sole fucosyltransferase in mice.^13^ Mono-fucosylated glycans (F) exhibited the highest abundance in the colonic mucin of mice on HF-NK and HF-BF fiber-supplemented diets. *O*-glycans terminating with galactose or N-acetylglucosamine (G) and glycans terminating with a single sulfate (GS) were also significantly more abundant in these diets. In contrast, *O*-glycans containing two Fuc residues (2F), a single Neu5Ac (N), or sulfate and Neu5Ac together (SN), were more abundant in NC and HF compared to both fiber supplemented diets (HF-NK and HF-BF). Previous studies have shown that dietary fiber inclusion can alter the total carbohydrate content of small intestinal mucins in rats.^25,26^ Our study goes further and provides detailed information on mucin glycosylation changes at the *O-*linked glycan structural level in response to diet.

The alteration of mucus layer glycosylation can have a profound impact on gastrointestinal health through its influence on innate immunity, inflammation status, and gut microbiota composition. The major gut mucin, MUC2, sequesters pathogens through the terminal display of Neu5Ac epitopes that act as binding targets and modulate adhesion.^44^ The continuous secretion and removal of the mucin containing mucus layer thus prevent the accumulation of microbes on the colorectal surface thereby reducing pathogen burden.^45^ Terminal residues such as sulfate and Neu5Ac have been shown to influence the structural integrity of the mucus layer.^45,46^ For example, the presence of negatively charged Neu5Ac residues determines the rheological properties of the mucus layer conferring rigidity and increased viscosity, ^46^ while the removal of terminal Neu5Ac is an initial step in the sequential degradation of mucin glycans. In this study, we observed a higher abundance of *O-*glycans containing one terminal sulfate (GS) in the HF-NK and HF-BF groups, while the abundance of glycans with Neu5Ac (N) and sulfate and Neu5Ac (SN) was higher in the NC and HF groups (Figure 4). This may suggest a diet-dependent functionality of glycans terminating with sulfate or Neu5Ac residues. Direct examination of mucus layer integrity and rigidity would be useful as a future study to establish the impact of diet on the functions of these *O-*glycans.

We then examined whether the concentration of SCFAs associates with the relative abundance of mucin *O-*glycans using correlation analysis. This identified significant positive correlations between glycans terminating with Neu5Ac (N) and two SCFAs, acetate, and butyrate (Pearson correlation = 0.55 and 0.36, respectively). Colonic mucin *O*-glycans have been proposed to regulate the production of SCFAs, ^47,48^ specifically the biosynthesis of butyrate.^47^ However, these previous studies were limited to quantifying the total mucin proteins and *O*-glycans. Our study provides further insight and demonstrates an association between specific mucin *O*-glycan structures and the concentrations of acetate and butyrate. Despite having a higher concentration of propionate in the HF-BF group (Figure S7), there were no statistically significant associations between the concentration of propionate and changes in mucin *O*-glycan terminal structures. Future studies on mucus layer thickness and MUC2 protein expression, which have been previously shown to vary based on dietary fiber intake, ^49,50^ will be useful in understanding whether total mucin *O*-glycans and consequent thickness of the mucin layer have an impact on the production of propionate and other SCFAs.

To our knowledge, we are the first to use modern glycomic techniques to show little change in the *O-*glycosylation of the colonic mucins with a high-fat diet, but with a clear and distinct effect of dietary fiber on the colonic mucus layer glycosylation. Our study lays the groundwork for a reliable and noninvasive mechanism to alter mucus layer glycosylation by dietary fiber supplementation that could become a powerful tool in improving gastrointestinal health by encouraging specific glycosylation patterns that may resist mucin degradation thereby affecting gut microbiota induced inflammation.

### Correlation network analysis identified specific associations between gut bacteria and mucin O-linked glycan structures

**Figure 5.**
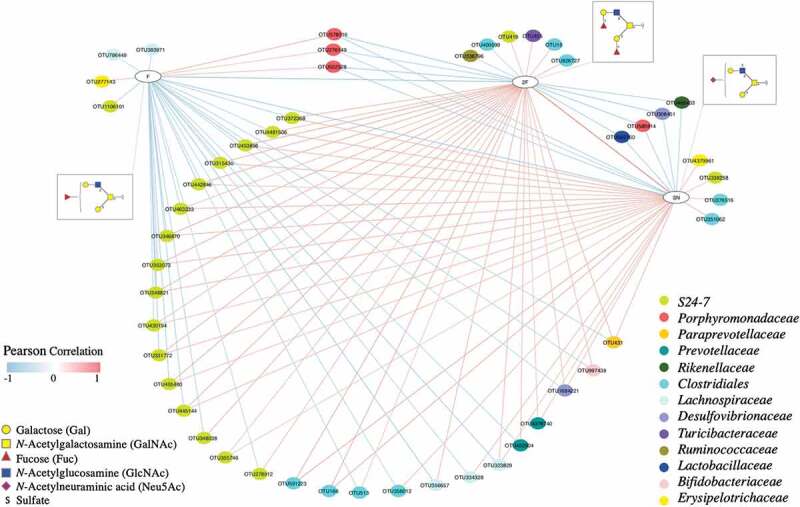
Network showing correlations between the relative abundance of bacterial OTUs and colonic mucin glycans. A pairwise Pearson correlation analysis was conducted between the relative abundance of OTUs and relative abundance of glycan groups. OTUs and glycan groups with significantly different abundances between dietary groups were included in the analysis. Only significant correlations (*P* < .05) were used to construct the network. Glycans are grouped based on the type and number of specific terminal structures: F- Fuc, 2F- double Fuc and SN- sulfate and Neu5Ac. The OTUs are shown in color-coded dots based on the bacterial family. A positive and negative correlation is presented by a red and blue line, respectively. The intensity of the color denotes the strength of the correlation.

Pairwise correlation analysis between the relative abundance of bacterial OTUs and relative abundance of specific glycan structures identified significant associations, which were used to construct a network consisting of 198 correlations and 83 nodes (Figure S8 and Table S7). We then generated subnetworks by extracting OTU nodes that directly connect to specific glycan terminal structures. According to these subnetworks, the relative abundance of OTUs within the families S24-7 and *Lachnospiraceae* and order *Clostridiales* showed a positive correlation with the abundance of glycan structures with two terminal Fuc residues (2F) and sulfate and Neu5Ac together (SN) (Figure 5). These OTUs negatively correlated with the abundance of glycans with a single Fuc (F) residue. Three OTUs in the family *Porphyromonadaceae* (*Parabacteroides distasonis*) were negatively correlated with glycan structures containing 2F and SN, and positively correlated with glycan structures with F.

The gut bacteria that positively associated with glycans with two Fuc residues (2F) were all negatively associated with glycans with one terminal Fuc (F). Previous studies have shown that the expression of terminal fucose to be gut microbiota-dependent, and its absence has been shown to significantly decrease the microbial alpha diversity and change the composition of the gut microbiota in both humans and mice.^51,52^ For example, mice that lack a functional copy of the α1-2 fucosyltransferase gene have a higher abundance of the genera *Parabacteroides, Eubacterium, Parasutterella, Bacteroides*, and family *Lachnospiraceae* and lower abundance of *Clostridiales*, indicating a link between the presence of terminal Fuc and the gut microbiota.^51^ Our study provides further insight into this by demonstrating a distinct association of glycans with one fucose and two fucose residues with the gut microbiota.

Specific gut bacteria utilize mucin *O*-glycans as an alternate energy source and degrade glycans through the use of proteases, sulfatases, and glycoside hydrolases (GH) encoded by their genomes.^3,21^ In this study, we observed several strong associations of bacterial OTUs in the species *Parabacteroides distasonis*, families S24-7 and *Lachnospiraceae*, and order *Clostridiales* with mucin *O*-glycans (Figure 5 and Figure S8). *Parabacteroides distasonis* and S24-7 are members of the phylum *Bacteroidetes* in which specific members have been reported to encode a range of GH enzyme families, including sialidase (GH33)^53^ and fucosidase (GH29), ^3,54^ and may have mechanisms to degrade mucin *O*-glycans. Several members in the order *Clostridiales* also have been reported to encode GH families, for example, sialidase (GH33)^55^ and galactosidase (GH98).^56^ While the observed correlations in our study could be due to the potential availability of such GH enzyme families in the specific OTUs and the ability to digest glycan structures, there are no direct investigations due to the current unavailability of culture isolates and individual genomes. Examinations of the individual bacterial genomes and biochemical analyses are crucial in validating the presence and functions of these GH families.

Regarding inter-glycan correlations, the relative abundance of glycans containing a single F was negatively correlated with glycans containing 2F and SN, and 2F glycans were positively correlated with SN (Figure 5). This may indicate a link between SN and F containing glycan structures; SN may either protect Fuc from digestion by specific bacterial groups or influence bacterial adhesion. This is in line with previous studies that have shown a protective effect of *O-*glycans with terminal sulfate and Neu5Ac on bacterial degradation of mucin glycans.^46,57^

At the interface between the host epithelium and luminal space, the mucus layer is critical in maintaining not only physical and chemical separation between the host and its microbiome but also in mediating host–microbiota interactions. As the main component of the intestinal mucus layers, mucins and their attached glycan epitopes serve as binding targets for gut bacterial adhesins, ^48^ provide an alternative carbon source for the gut microbiota^3,4^ and influence microbial diversity through the application of selective pressures.^51,52^ Conversely, gut bacteria and their metabolites can regulate colonic mucin *O*-glycosylation through modulating the expression of host glycosyltransferases.^12,13^ Our results suggest a relationship between the abundance of specific gut bacteria and individual colonic mucin glycan structures. These correlations could potentially affect three interactions: 1) host glycan-mediated selection of bacteria, 2) bacterial degradation of glycans, and 3) bacterial-induced modulation of host glycosylation. These associations suggest new avenues to perform a comprehensive analysis of the interactions between the gut microbiota and intestinal mucus layer. Experiments specifically targeting glycosylation, such as binding assays between specific colonic mucin glycans and individual gut bacteria, will be useful in elucidating the biological mechanisms of these correlations. Our results suggest that modulation of colonic mucin glycosylation could provide a targeted mechanism to modify the gut microbiota composition.

## Conclusions

High-fat diet consumption significantly altered the gut microbiota, SCFA production, and host physiology compared to the normal chow-fed group, however, only minor differences were observed in colonic mucin *O*-glycosylation profiles. Conversely, dietary fiber addition resulted in dramatic changes in mucin *O*-glycosylation profiles, and significant increases in propionate production and the abundance of known fiber-digesting gut bacteria. Our results suggest a distinct and a greater impact of dietary fiber on colonic mucin *O-*glycosylation than high-fat diet alone. Correlation network analysis revealed associations between individual colonic mucin glycans and specific gut bacteria. This is the first study to provide comprehensive insight into the response of colonic mucin *O-*glycosylation toward dietary interventions and how individual colonic mucin glycan structures associate with specific gut bacteria. The intimate affiliation between gut bacteria and mucin glycan structures that line the colon surface is critical yet hugely unexplored. Our study suggests that coupling gut microbiota studies and glycomic mucin analysis should become a more common practice in the field of gut microbiota research.

## Materials and methods

### Animal handling and sample collection

All experimental procedures were approved by the Animal Ethics committees at the University of Sydney (2014/611), Australia and Macquarie University (5201500129), Australia.

The two fiber products, NutriKane™ and Benefiber®, are derived from dried whole sugarcane stem and wheat dextrin, respectively. NutriKane was produced and provided by Gratuk Technologies Pty Ltd, Australia. Benefiber, produced by GlaxoSmithKline, Australia, was purchased from a local Australian supermarket. All experimental diets were produced by Specialty feeds, WA, Australia. Custom-made high-fat diets with fiber additions contained 4.7% (w/w) of either NutriKane or Benefiber as a replacement of 4.7% (w/w) cellulose in the high-fat diet. Nutritional information and ingredients of experimental diets are provided in Table S1.

A total of 50, 11-week old male C57BL/6J mice (Animal Resource Center, WA, Australia) were cohoused (two per cage) under monitored temperature (20–26°C), humidity (40–60%), light and dark cycle (12 hr-12 hr) and with *ad libitum* access to water and feed during the experiment. Following a two-week acclimatization on a normal chow (14 kJg^−1^, 12% of total energy from fat), mice were randomized into two groups based on the body weight and were fed either the normal chow (n = 9) or a high-fat diet (24.0 kJg^−1^, 81% of total energy from fat). After 17 weeks, the high-fat diet group was further randomized into three groups based on the body weight and area under the curve of an intraperitoneal glucose tolerance test. These groups were fed a high-fat diet (n = 14), high-fat diet modified with NutriKane (n = 14) or high-fat diet modified with Benefiber (n = 13) for further 15 weeks; details of the experimental design are provided in Figure S1.

Intraperitoneal glucose tolerance tests (IPGTT) were performed at weeks 17 and 23. Mice were fasted for six hours during the light cycle. Blood glucose levels were measured from the tail vein using a Freestyle Lite blood glucose monitoring system (Abbott Pty Ltd, Australia) prior to intraperitoneally injecting glucose (2.0 gkg^−1^). Blood glucose levels were measured at 15, 30, 45, 60, 90, and 120 minutes after injection.

Individual body weight and food intake per cage were measured weekly. Fecal samples were collected aseptically at week 0, 17, 23, and 32 and stored at −80°C prior to subsequent microbiota and metabolite analyses.

Blood (100 µL) was collected from the mandibular vein at week 0, 17, and 32. Samples were gently mixed with EDTA at a final concentration of 4 mM, pH 7.0. After separating the plasma through centrifugation at 1000 x g for 10 min at 4°C, it was stored  at −80°C before analysis.

Mice were perfused with ice-cold 1X phosphate-buffered saline at week 32, the liver samples were immediately snap-frozen in dry ice and stored at −80°C before subsequent analysis. Mice were euthanized by cervical dislocation.

### Quantification of circulating plasma biomarkers

Plasma cytokines and markers of diabetes and obesity were quantified using Bio-Plex Pro™ mouse cytokine (M60000007A) and diabetes (171F7001M) assay kits, respectively, according to the manufacturer’s instructions (Bio-Rad, Australia). The statistical analysis was performed using a Student’s t-test and ANOVA (Wald Chi-square test), *P*-value correction was performed according to the Holm’s method.

### Mass spectrometry-based proteomics analysis

Perfused liver (10 mg) was subjected to LC-MS/MS analysis employing SWATH-based proteomics as the quantitation strategy. Library and SWATH-MS data were acquired in a 6600 TripleTOF MS coupled with an Ekspert 415 LC system (Sciex, Australia). Database searches included trypsin as the digestion enzyme and carbamidomethylation as the fixed cysteine modification, reversed database search was enabled to allow false discovery rate (FDR) calculation, and protein global FDR was established at 1%. Information was extracted from SWATH-MS peak areas using Peak View version 2.1 with SWATH MicroApp 2.0 (Sciex, Australia) using the library search output file. Perseus (version 1.5.5) was employed for statistical analysis.

### 16S rRNA gene amplicon sequencing and bioinformatic analysis

Total community DNA was isolated from fecal samples collected at week 0, 17, 23, and 32 using a FastDNA spin kit (MP Biomedicals, Australia) according to the manufacturer’s instructions. The V4 region of the 16S rRNA gene was amplified using Five prime hot master mix (VWR, Australia) with 515 F (5ʹ-GTGCCAGCMGCCGCGGTAA-3ʹ) and 806 R (5ʹ-GGACTACHVGGGTWTCTAAT-3ʹ) primers with custom barcodes. The resulting amplicons were quantified (Quant-iT™ PicoGreen® Invitrogen, Australia), equal molar amounts of barcoded amplicons from each sample were pooled, gel purified using a Wizard® SV gel and PCR clean up system (Promega, Australia) and sequenced using an Illumina MiSeq platform (2 x 250 bp, paired-end sequencing) at the Ramaciotti Centre for Genomics, Australia.

Demultiplexed raw sequence data were processed using Quantitative Insights Into Microbial Ecology (QIIME) software (version 1.9.1), ^58^ and operational taxonomic units (OTUs) were determined at 97% similarity using an open-reference protocol against the Greengenes (version 13_8) database.^59^ A total of 18,527,820 reads were sequenced from the 200 samples (mean 89,997 ± 28,063). OTUs with less than 0.005% reads were filtered out and reads per sample were rarefied at 44,361 reads prior to further statistical analyses.

Statistical analysis of the gut microbiota sequencing data was conducted using the PRIMER-7 software package.^60^ Non-metric multidimensional scaling (nMDS) plots were constructed based on Bray–Curtis similarity matrices of Log (x + 1) transformed abundance of the OTUs. Permutational Multivariate Analysis of Variance (PERMANOVA) tests with 9999 permutations were conducted to investigate differences in the microbial community structure. Distinct phylotypes (families and OTUs) between dietary groups were identified using the Linear discriminant analysis (LDA) effect size (LEfSe) method (online Galaxy version 1.0)^61^ using default parameters. The dietary groups were used as the classes of subjects (no subclasses).

### Quantification of SCFAs

The concentration of SCFAs (acetate, propionate, and butyrate) was quantified using fecal samples collected at weeks 17, 23, and 32. Approximately 20–50 mg of feces was extracted with 500 µL of 70% (v/v) ethanol and 0.1% (v/v) trifluoroacetic acid (TFA) solution spiked with an internal standard (4-methyl valeric acid) at a final concentration of 100 ppm. The solution was mixed thoroughly, then centrifuged at 14,000 x g at 4°C for 30 minutes to pellet the fecal material. The top 200 µL was removed and analyzed using a Shimadzu GC-17A gas chromatograph with a flame ionization detector (GC-FID, Shimadzu GC-17A). Samples were separated on a 30 m x 0.25 × 0.5 µm i.d. HP-INNOWax fused silica column (Hewlett-Packard, Australia) as per the manufacturer’s instructions. GC-FID analysis for each sample was performed in three technical replicates (n = 450). All measurements were normalized for the weight of fecal samples used for SCFA quantification.

### Mucin extraction and O-glycan characterization

Mucus was obtained by vacuum suction from the entire length of the colon. Colonic mucins were obtained from the mucus by precipitation with GuHCl. *O-g*lycans were released by reductive β-elimination and subjected to PGC-LC-ESI-MS/MS analysis according to a previously established protocol.^62^ *O*-glycans were separated on an Agilent 1100 LC system coupled to an Agilent 6330 ESI-MS (Agilent Technologies, Inc., USA). Glycan compositions were calculated from the mass using GlycoMod (https://web.expasy.org/glycomod/) and glycan structures were assigned by manual interpretation of the tandem MS fragmentation spectra. Glycan peaks were quantified by relative abundance using Skyline (version 3.7.0.11317, MacCoss Lab, USA) for assisted peak picking, generation of extracted ion chromatograms (EICs), and integration of EIC peak areas. Glycan structures were drawn using GlycoWorkbench 2 (version 2.1) using SNFG nomenclature.

### Statistical analysis

D’Agostino-Pearson normality tests were performed, Kruskal–Wallis test with Dunn’s multiple comparison test or Tukey’s multiple comparison test was used where appropriate to determine statistically significant differences. Significant differences in body weight, feed intake, IPGTT, concentration of biomarkers, organ weight, microbiome alpha diversity, SCFA concentration, and glycan abundance were determined through comparison between the dietary groups using GraphPad Prism (version 8.4) software (GraphPad Software, USA).

Pairwise correlation analyses were conducted between significantly differentially abundant bacterial OTUs and concentration of each of the three tested SCFAs, and the relative abundance of colonic mucin glycans and concentration each SCFA. Pearson correlation coefficients were determined using the Hmisc R package.^63^ Correlations with a *P* value > .05 were excluded from further analysis.

### Correlation network analysis

Pairwise correlation analyses between significantly differentially abundant gut bacterial OTUs and colonic mucin glycans were performed using their relative abundances. Pearson correlation coefficients were determined using Hmisc R package.^63^ The OTUs and glycans with significantly different abundances between dietary groups were used for this analysis. OTU-OTU correlations and correlations with a *P* value > .05 were excluded from further analysis. Correlation networks were constructed using Cytoscape software (version 3.6.1).

## Supplementary Material

Supplemental Material

## Data Availability

The 16S rRNA gene sequence data generated and analyzed during the current study are available in the GenBank Sequence Read Archive database under accession number PRJNA532979. The proteomics data generated and analyzed during the current study are available in the PRIDE database under accession number PXD014132. reviewer07713@ebi.ac.uk
