# Supplementary material for: Changes in dietary fiber intake in mice reveal associations between colonic mucin O-glycosylation and specific gut bacteria
Source: Gut Microbes. 2020 Sep 29;12(1):1802209. doi: 10.1080/19490976.2020.1802209 (PMC7781582; doi:10.1080/19490976.2020.1802209)
Supplement: Supplemental Material [file KGMI_A_1802209_SM5248.zip › Supplementary information/Supplementary Figure and Table legends.docx]

**Supplementary Figure and Table legends**

**Figure S1** The experimental design. **(A)** The timeline of the experiment, 50, C57BL/6 mice were randomised into one of two dietary groups, normal chow (NC) or high fat diet (HF) at week 0. After 17 weeks, a subset of the HF group was further randomised into, high fat modified with NutriKane (HF-NK) or high fat modified with Benefiber (HF-BF) diets. The time and type of samples/measurement collected are indicated. **(B)** The nutrient composition of each diet. Nutritional information and ingredients of experimental diets are provided in Table S1.

**Figure S2** Glucose tolerance, plasma biomarker and liver protein expression. Data shown for mice fed normal chow (NC), high fat diet (HF), HF modified with NutriKane (HF-NK) and HF modified with Benefiber (HF-BF). **(A)**Area under the curve (AUC) of IPGTT conducted at week 23. The concentration of **(B)** GLP-1, **(C)** Insulin, **(D)** PAI-1, **(E)** IL-1β and **(F)** GM-CSF expressed as mean of Log(2) normalised fluorescence intensity with ± SD (**P*< 0.05, ***P*< 0.01). **(G)** Mass Spectrometry-based hepatic protein expression of mice in each dietary group. Each column represents a single mouse. Hierarchical clustering was performed based on ANOVA multiple sample test and permutation-based FDR 0.05 on the normalised Z-score intensity values. Up- and down-regulation of proteins are denoted by red and green, respectively.

**Figure S3** Plasma concentration of markers of inflammation (IL-2, IL-4, IL-5, IL-10, IFN gamma and TNF alpha), and diabetes and obesity (leptin, glucagon, GIP, ghrelin and resistin). The concentration of these markers had no statistically significant difference between the four treatment groups. Data is shown as mean of Log(2) normalised fluorescence intensity with ± SD. Blood samples for the analysis were collected at week 0, 17, and 32. NC: normal chow, HF: high fat diet, HF-NK: high fat diet with NutriKane and HF-BF: high fat diet with Benefiber.

**Figure S4** Mass of the organs excised from mice at week 32. The mass of the caecum, epididymal white adipose tissue, liver, kidneys and ratio between epididymal white adipose tissue mass and body weight per mouse is shown (mean ± SD). Significance (* *P* < 0.05) was determined using a Mann-Whitney test.

**Figure S5** Average weekly **(A)** feed intake **(B)** energy intake and **(C)** body weight per mouse. Mean values per dietary group with ± SD are shown. Significance was determined based on Tukey’s multiple comparison tests, (*****P* < 0.0001) compared to the normal chow group.

**Figure S6** Key bacterial families of the gut microbiota responding to dietary changes at week 32. Data was obtained using LEfSe analyses between **(A)** HF vs NC, **(B)** HF vs HF-NK and **(C)** HF vs HF-BF groups. The left histogram shows the LDA scores computed for each bacterial family. The unpaired heatmap on the right shows the relative abundance (Log_10_ transformation) of the families in each mouse. In the heat map, rows correspond to bacterial families and columns correspond to individual mice in each dietary group. Blue and black denote the highest and lowest relative abundance, respectively, as shown in the legend. **(D)** The relative abundance (%) of these key bacterial families at week 0, 17, 23 and 32.

**Figure S7** Fecal concentration (mmolL^-1^g^-1^) of **(A)** acetate, **(B)** butyrate and **(C)** propionate. Data is shown per mouse in each dietary group at week 17, 23 and 32. Mean values with ± SD are shown (**** *P* < 0.0001, *** *P* < 0.001, ** *P* < 0.01 and * *P* < 0.05). The concentrations per mouse are provided in Table S4.

**Figure S8** A network of pairwise correlations between the relative abundance of bacterial OTUs and colonic mucin glycans. Pairwise Pearson correlation analysis was conducted between the relative abundance of OTUs and relative abundance of glycan groups, OTUs and glycan groups with significantly different abundances between dietary groups were included in the analysis. Only the significant correlations (*P* < 0.05) were used to construct the network. Glycans are grouped based on the type and number of terminal structures: G- Gal, F- Fuc, 2F- double Fuc, GS-sulfate and Gal, N-Neu5Ac, 2N- double Neu5Ac, SN- sulfate and Neu5Ac, SF- sulfate and Fuc, FN- Fuc and Neu5Ac, and SFN- sulfate, Fuc and Neu5Ac. The OTUs are shown in colour-coded dots based on the bacterial family. A positive and negative correlation is presented by a red and blue line, respectively. The intensity of the colour denotes the strength of the correlation.

**Table S1** Nutritional information and ingredients of experimental diets. **(A)** Composition of normal chow and high fat diet. Cellulose (4.7% w/w) in the high fat diet was replaced with NutriKane or Benefiber to produce HF-NK and HF-BF diets, respectively. **(B)** Ingredients and nutritional information of NutriKane and Benefiber.

**Table S2** Significantly regulated proteins expressed as **(A)** NC/HF, **(B)** HF-NK/HF, **(C)** HF-BF/HF and ratios. Two-sample t-test (*P* < 0.05), fold change>1.5.

**Table S3** The relative abundance of bacterial OTUs that were found to be significantly differentially abundant between dietary groups at week 32. Data was obtained based on LEfSe analyses between NC vs HF, HF vs HF-NK and HF vs HF-BF groups. LEfSe analyses were performed with the following parameters, Kruskal-Wallis test among classes (*P*< 0.05), Wilcoxon test between classes (*P* < 0.01) and the threshold on the logarithmetic LDA score for discriminative features > 3.0.

**Table S4** Concentration of acetate, butyrate and propionate in each treatment at week 17, 23 and 32. Measurements per mouse in each dietary group are provided.

**Table S5** The Pearson correlation coefficient (*R*) of pair-wise correlation comparisons between the relative abundance of gut bacteria and short chain fatty acids. Only significant correlations are shown (*P* < 0.05).

**Table S6** Composition and structure of *O-*glycans from colonic MUC2. NC: standard diet, HF: high fat diet, HF-NK: high fat diet modified with NutriKane, HF-BF: high fat diet modified with Benefiber. Values provided are in % relative abundance with the standard deviation (SD).

**Table S7** The Pearson correlation coefficient (*R*) of pair-wise correlation comparisons between the relative abundance of gut bacteria and colonic glycans. Only the significant correlations are shown (*P* < 0.05). NC: standard diet, HF: high fat diet, HF-NK: high fat diet modified with NutriKane, HF-BF: high fat diet modified with Benefiber.
