# Supplementary material for: Changes in dietary fiber intake in mice reveal associations between colonic mucin O-glycosylation and specific gut bacteria
Source: Gut Microbes. 2020 Sep 29;12(1):1802209. doi: 10.1080/19490976.2020.1802209 (PMC7781582; doi:10.1080/19490976.2020.1802209)
Supplement: Supplemental Material [file KGMI_A_1802209_SM5248.zip › Supplementary information/Table S6.pdf]

Table S6 Composition and structure of *O*-glycans from colonic Muc2. NC: standard diet, HF: high fat diet, HF-NK: high fat diet modified with NutriKane, HF-BF: high fat diet modified with Benefibre. Values provided are in % relative abundance with the standard deviation (SD).

Mass (m/z)			Composition	Core	Proposed structure*	NC	HF	HF-NK	HF-BF
$[M-H]^-$	$[M-H]^{2-}$	[M]				Mean ± SD	Mean ± SD	Mean ± SD	Mean ± SD
530		531	(Hex)1 (HexNAc)1 (Deoxyhexose)1	1	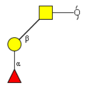	12.6 ± 4.4	12.0 ± 6.6	19.0 ± 7.4	15.8 ± 9.1
587		588	(Hex)1 (HexNAc)2	2	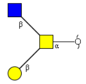	5.4 ± 3.2	5.3 ± 3.9	14.3 ± 8.1	11.6 ± 8.3
667		668	(Hex)1 (HexNAc)2 (Sulph)1	2	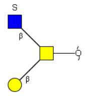	4.5 ± 2.6	2.5 ± 2.4	8.1 ± 4.3	9.7 ± 7.4
733a		734a	(Hex)1 (HexNAc)2 (Deoxyhexose)1	3	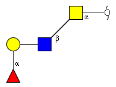	2.0 ± 2.6	1.9 ± 3.2	4.2 ± 4.3	2.6 ± 2.6
733b		734b	(Hex)1 (HexNAc)2 (Deoxyhexose)1	2	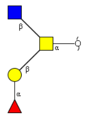	6.4 ± 4.6	13.9 ± 5.4	15.5 ± 7.1	21.0 ± 10.2
749		750	(Hex)2 (HexNAc)2	2	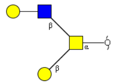	1.2 ± 1.2	0.1 ± 0.2	0.5 ± 0.8	0.0 ± 0.0
790		791	(Hex)1 (HexNAc)3	2	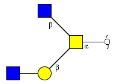	0.5 ± 0.7	0.2 ± 0.4	0.3 ± 0.7	0.5 ± 0.7
813		814	(Hex)1 (HexNAc)2 (Deoxyhexose)1 (Sulph)1	2	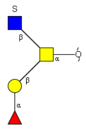	11.7 ± 7.9	13.9 ± 7.6	19.8 ± 11.3	20.6 ± 8.0

829	830	(Hex)2 (HexNAc)2 (Sulph)1	2	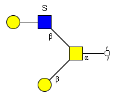	1.4 ± 2.0	0.2 ± 0.4	0.2 ± 0.5	0.2 ± 0.5
895a	896a	(Hex)2 (HexNAc)2 (Deoxyhexose)1	2	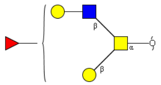	1.9 ± 1.1	2.8 ± 1.7	0.8 ± 1.9	1.1 ± 1.9
895b	896b	(Hex)2 (HexNAc)2 (Deoxyhexose)1	2	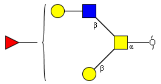	2.9 ± 2.1	1.3 ± 1.5	0.4 ± 1.1	1.2 ± 1.9
936	937	(Hex)1 (HexNAc)3 (Deoxyhexose)1	4	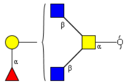	1.3 ± 1.9	1.4 ± 1.2	0.0 ± 0.0	0.5 ± 0.8
975a	976a	(Hex)2 (HexNAc)2 (Deoxyhexose)1 (Sulph)1	2	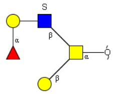	4.2 ± 3.2	3.1 ± 2.8	0.4 ± 0.4	0.6 ± 0.8
975b	976b	(Hex)2 (HexNAc)2 (Deoxyhexose)1 (Sulph)1	2	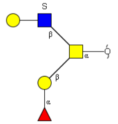	0.9 ± 1.2	1.2 ± 1.5	0.8 ± 1.2	0.0 ± 0.0
1040a	1041a	(Hex)2 (HexNAc)2 (NeuAc)1	2	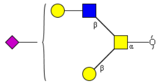	0.9 ± 1.4	0.0 ± 0.1	0.0 ± 0.0	0.0 ± 0.0
1040b	1041b	(Hex)2 (HexNAc)2 (NeuAc)1	2	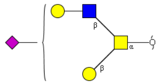	4.6 ± 2.1	4.5 ± 3.8	0.0 ± 0.1	0.1 ± 0.2
1041	1042	(Hex)2 (HexNAc)2 (Deoxyhexose)2	2	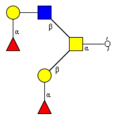	9.8 ± 4.1	10.3 ± 5.3	0.1 ± 0.3	0.2 ± 0.4

1041		1042	(Hex)2 (HexNAc)2 (Deoxyhexose)2	2	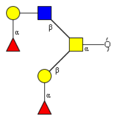	0.7 ± 0.8	1.3 ± 1.5	0.0 ± 0.0	0.1 ± 0.1
1120a		1121a	(Hex)2 (HexNAc)2 (NeuAc)1 (Sulph)1	2	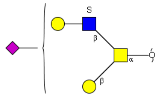	1.4 ± 1.5	0.4 ± 1.0	0.0 ± 0.0	0.1 ± 0.3
1120b		1121b	(Hex)2 (HexNAc)2 (NeuAc)1 (Sulph)1	2	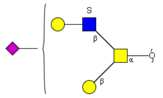	7.0 ± 3.6	9.0 ± 4.7	1.1 ± 0.8	1.4 ± 1.0
1121		1122a	(Hex)2 (HexNAc)2 (Deoxyhexose)2 (Sulph)1	2	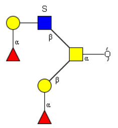	0.3 ± 0.4	0.1 ± 0.2	0.0 ± 0.1	0.1 ± 0.2
	560	1122b	(Hex)2 (HexNAc)2 (Deoxyhexose)2 (Sulph)1	2	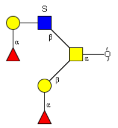	0.3 ± 0.3	0.2 ± 0.4	0.0 ± 0.0	0.0 ± 0.0
1187a		1188	(Hex)2 (HexNAc)2 (Deoxyhexose)1 (NeuAc)1	2	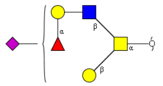	0.9 ± 0.6	0.9 ± 0.7	0.0 ± 0.1	0.2 ± 0.3
1187b		1188	(Hex)2 (HexNAc)2 (Deoxyhexose)1 (NeuAc)1	2	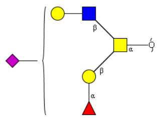	1.0 ± 0.6	1.0 ± 1.0	0.0 ± 0.0	0.0 ± 0.0
1245a		1246a	(Hex)2 (HexNAc)3 (Deoxyhexose)2	4	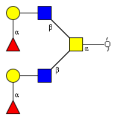	0.8 ± 0.9	1.0 ± 1.1	0.2 ± 0.6	0.0 ± 0.1

1245b		1246b	(Hex)2 (HexNAc)3 (Deoxyhexose)2	4	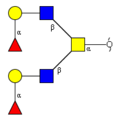	2.9 ± 2.7	1.2 ± 0.8	0.0 ± 0.0	0.1 ± 0.2
1266		1267	(Hex)2 (HexNAc)2 (Deoxyhexose)1 (NeuAc)1 (Sulph)1	2	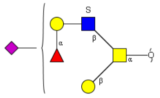	0.9 ± 0.5	0.8 ± 0.6	0.0 ± 0.0	0.1 ± 0.2
1332		1333	(Hex)2 (HexNAc)2 (NeuAc)2	2	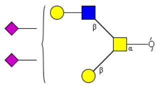	0.9 ± 1.0	0.7 ± 0.7	1.5 ± 0.9	1.7 ± 1.8
1390		1391	(Hex)2 (HexNAc)3 (Deoxyhexose)1 (NeuAc)1	2	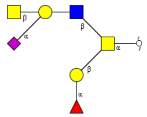	0.5 ± 0.5	1.0 ± 0.7	1.0 ± 0.8	1.3 ± 1.6
	734	1470	(Hex)2 (HexNAc)3 (Deoxyhexose)1 (NeuAc)1 (Sulph)1	2	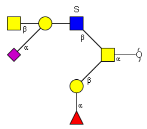	7.0 ± 3.0	2.3 ± 0.8	7.3 ± 5.9	4.0 ± 3.0
	766	1535	(Hex)2 (HexNAc)3 (NeuAc)2	2	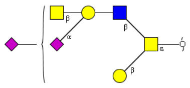	0.4 ± 0.6	0.1 ± 0.3	2.8 ± 1.7	1.5 ± 1.5
	840a	1682a	(Hex)2 (HexNAc)3 (Deoxyhexose)1 (NeuAc)2	2	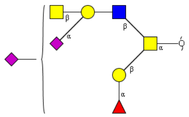	1.5 ± 1.8	1.8 ± 1.5	0.0 ± 0.0	2.2 ± 1.6
	840b	1682b	(Hex)2 (HexNAc)3 (Deoxyhexose)1 (NeuAc)2	2	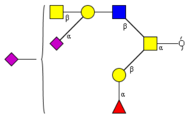	0.5 ± 0.7	0.4 ± 0.4	0.3 ± 0.4	0.2 ± 0.2
	868	1739	(Hex)2 (HexNAc)4 (NeuAc)2	2	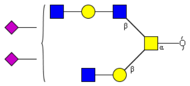	0.1 ± 0.3	0.0 ± 0.0	0.1 ± 0.3	0.1 ± 0.2

884	1770	(Hex)3 (HexNAc)4 (NeuAc)1 (Sulph)2	2	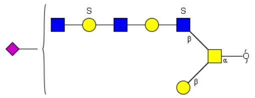	1.5 ± 1.8	0.9 ± 0.8	0.4 ± 0.8	0.4 ± 0.3
941	1885	(Hex)2 (HexNAc)4 (Deoxyhexose)1 (NeuAc)2	2	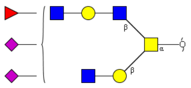	0.6 ± 1.2	0.3 ± 0.2	0.0 ± 0.0	0.0 ± 0.0
957	1916	(Hex)3 (HexNAc)4 (Deoxyhexose)1 (NeuAc)1 (Sulph)2	2	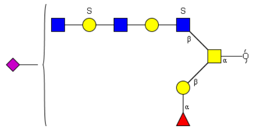	0.2 ± 0.4	0.4 ± 0.6	0.0 ± 0.0	0.4 ± 0.7

*Proposed structures were based on comparison with the structures reported in previous studies (Arike et al., 2017; Thomsson et al., 2012). Hex = glucose or galactosamine, HexNAc = N-Acetylglucosamine or N-Acetylgalactosamine, NeuAc = N-Acetylneuraminic acid, Deoxyhexose = Fucose, Sulph = Sulphate.
